# Supplementary material for: Genomic adaptation of the picoeukaryote Pelagomonas calceolata to iron-poor oceans revealed by a chromosome-scale genome sequence
Source: Commun Biol. 2022 Sep 16;5:983. doi: 10.1038/s42003-022-03939-z (PMC9481584; doi:10.1038/s42003-022-03939-z)
Supplement: Supplementary file 2 — Supplementary Information [file 42003_2022_3939_MOESM2_ESM.pdf]

Supplementary information

Genomic adaptation of the picoeukaryote *Pelagomonas calceolata* to iron-poor oceans revealed by a chromosome-scale genome sequence.

Nina Guérin[1,2], Marta Ciccarella[1], Elisa Flamant[1,2], Paul Frémont[1,2], Sophie Mangenot[1,2], Benjamin Istace[1], Benjamin Noel[1], Caroline Belser[1], Laurie Bertrand[1,2] , Karine Labadie[2,3], Corinne Cruaud[2,3], Sarah Romac[4], Charles Bachy[4,5], Martin Gachenot[5], Eric Pelletier[1,2], Adriana Alberti[1,2,6], Olivier Jaillon[1,2], Patrick Wincker[1,2], Jean-Marc Aury[1], Quentin Carradec[1,2]*

*Correspondence and requests for materials should be addressed to qcarrade@genoscope.cns.fr

Supplementary Figures

Figure S1: Assembly and duplicated regions of the *P. calceolata* genome. 2

Figure S2: Hi-C interaction map for *P. calceolata* genome. 3

Figure S3: Identification of introner elements from repeat families. 4

Figure S4: *P. calceolata* proteins shared with other stramenopiles. 5

Figure S5: Estimation of the relative abundance of P. calceolata with 2 different methods. 6

Figure S6: Relative abundance and ecological niche of *P. calceolata* in the 0.8-2000 µm size-fraction. 7

Figure S7: Modelled genomic relative abundance of *P. calceolata* in present day and at the end of the century. 8

Figure S8: Expression levels of genes involved in iron transport and coding for superoxide dismutases. 9

Figure S9: Expression levels of genes involved in nitrogen compounds uptake. 10

Figure S10: Expression levels of genes involved in nitrogen metabolism in *P. calceolata.* 11

Figure S11: Nitrate and nitrite sensing proteins in *P. calceolata* and other eukaryotes. 12

Supplementary Notes

Supplementary Note 1: Duplicated regions in *P. calceolata* genome 13

Supplementary Note 2: Description of the Hi-C result 13

Supplementary Note 3: Introns and Introner Elements 13

Supplementary Note 4: GC content distribution along *P. calceolata* chromosomes 14

Supplementary Note 5: Centromeres, recombination and meiosis in *P. calceolata* 15

Supplementary References 16

a **b**

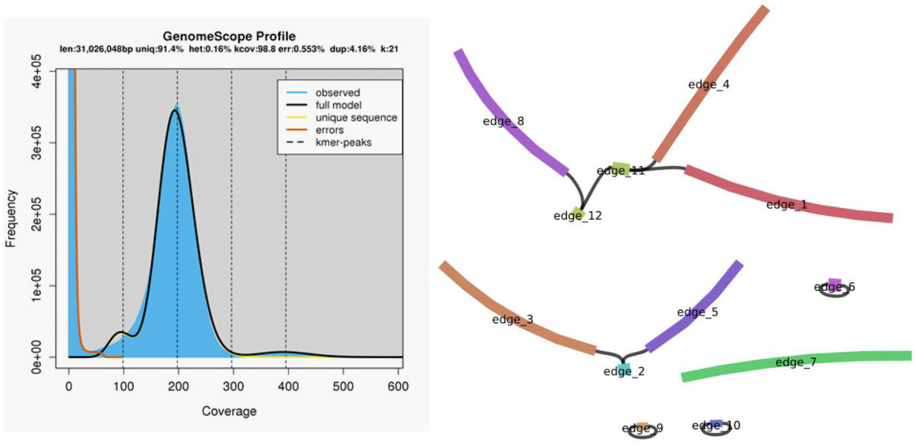

c

Contig	Edge path	Size	Coverage
1	11,1,*	6 277 107	246
2	*,7,*	6 137 160	240
3	*,-2,3,*	5 383 939	241
4	*,12,8,*	5 311 685	243
5	11,4,*	5 287 858	244
6	*,5,2,*	3 907 979	243
7	10	90 791	27
8	9	39 491	78

d

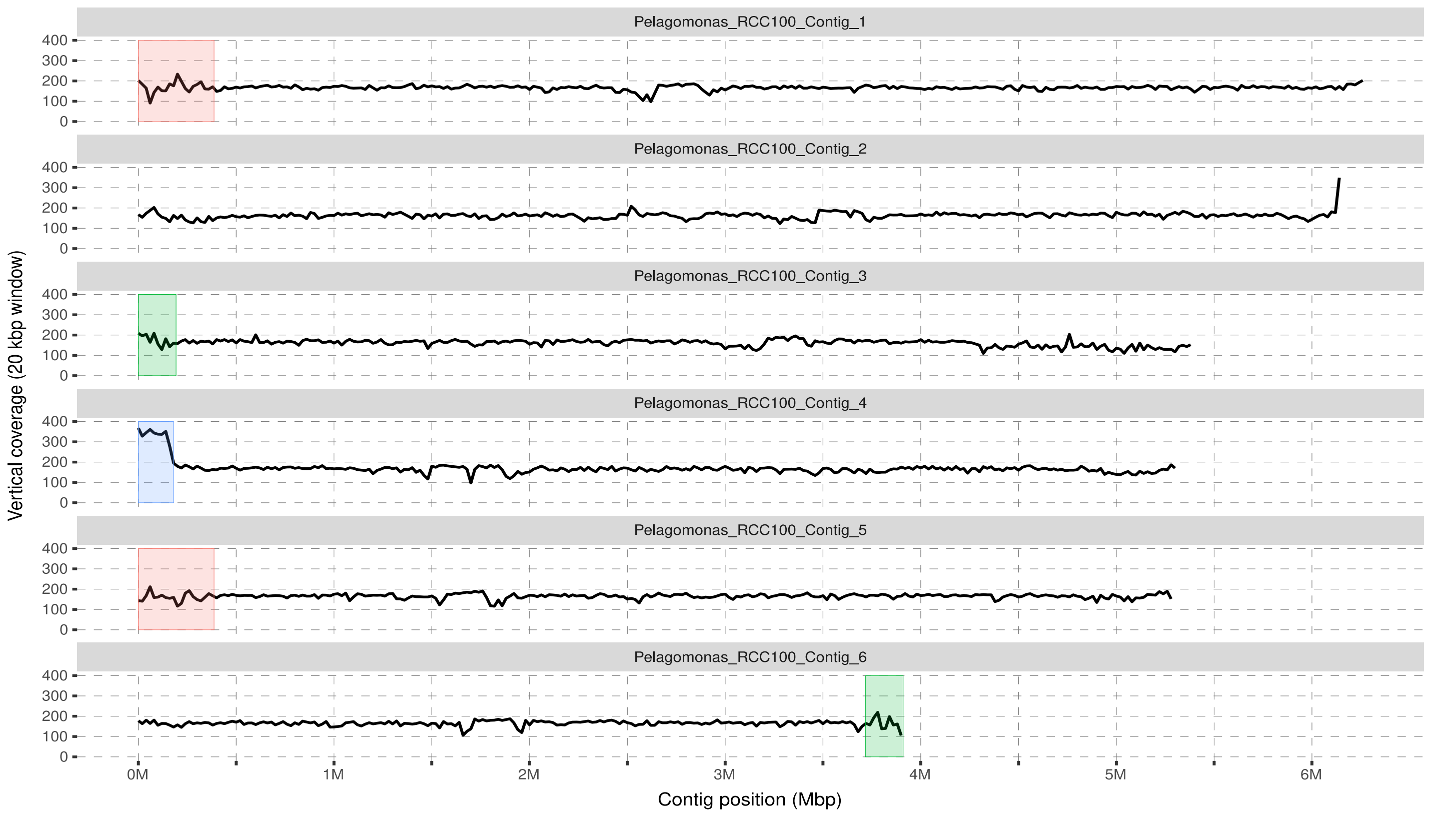

Figure S1: Assembly and duplicated regions of the *P. calceolata* genome. a) K-mer profile of *P. calceolata* Illumina reads.The graph shows the fit of the GenomeScope model (black) to the observed k-mer frequencies (blue). The k-mers presenting high frequency and low coverage (red) are considered as sequencing errors. b) Graphical representation of the Flye assembly graph generated with Bandage tool (Wick et al. 2015). Each colored box represents a sequence (edge) of the assembly. Edges connected with one or several black lines indicate unresolved repeats at the extremity of the contig (e.g edge_2 is connected to edge_3 and/or edge_5). Based on the vertical coverage, Flye chose to duplicate edge_2 to form contig 3 and contig 6). c) Table of the assembly paths and metrics of the assembled contigs detected by Flye. Numbers in the edge path column correspond to the edges in panel A. Telomeric repeats located at the ending edges of the assembly graph are reported as a star in the path column. d) Vertical coverage of Illumina reads in the P. calceolata genome. The dark line representes the number of Illumina short reads mapped over a window of 20 Kb on the 6 nuclear contigs of P. calcolata. Red and green boxes indicate highly similar regions (>99% of identity). The instability of the read coverage in these regions may be explained by the presence of variants in one of the two copies. The blue box is the only large region with a vertical coverage twice as high as the genome average.

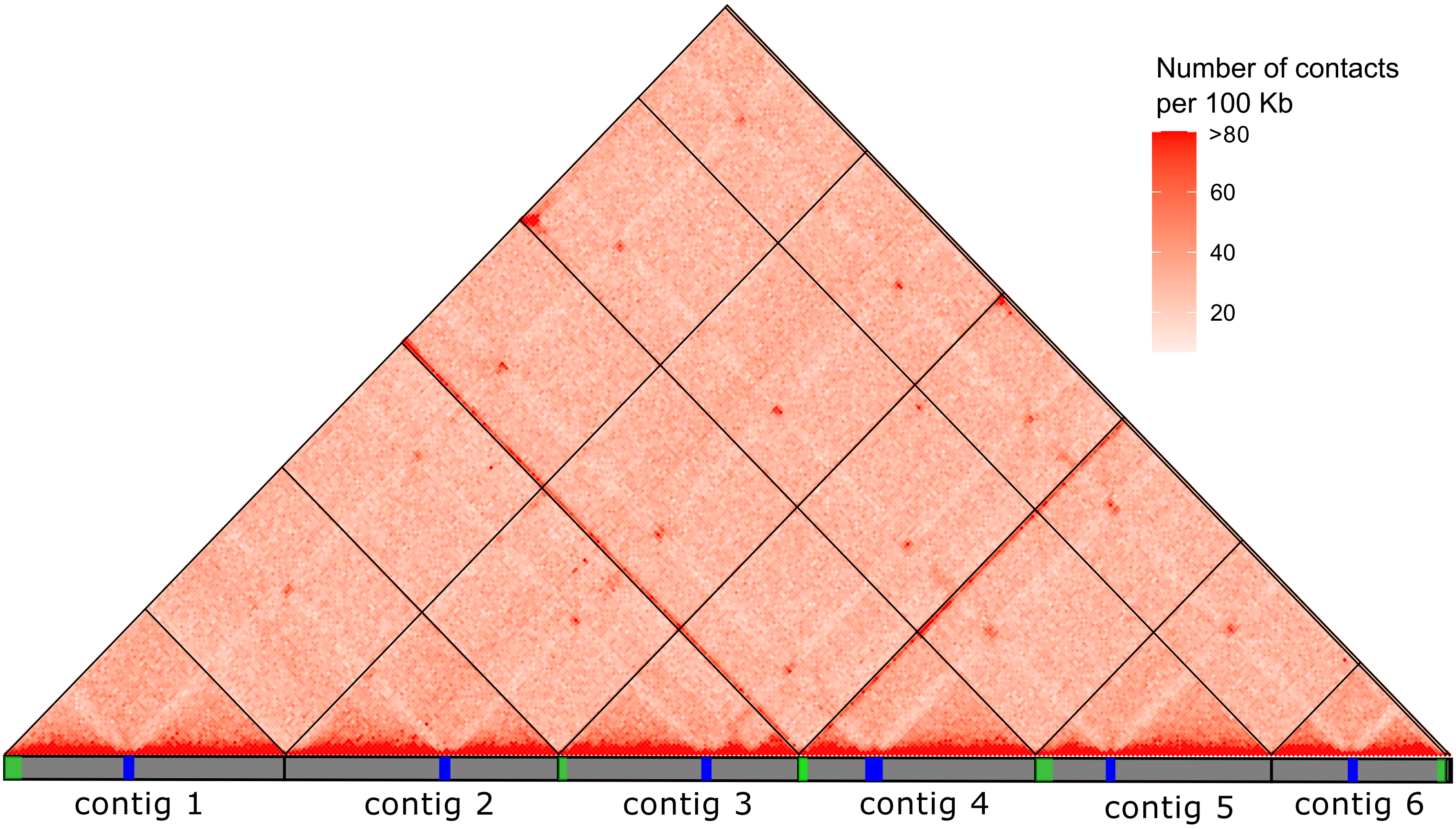

Figure S2: Hi-C interaction map for *P. calceolata* genome. The localisation of interaction points in the *P. calceolata* genome sequence are indicated by red dots (window of 100Kb). The red intensity is proportional to the number of reads covering this interaction. The low-GC region in each contig is indicated in blue. Red dots corresponding to these regions indicate physical proximity between centromeres. Duplicated regions are indicated in green. Red dots corresponding to these regions are due to the random aligment of reads on the dupliacted regions. The interaction background is more intense at the beginning of contig 4 because this duplicated region was collapsed in the assembly resulting in a double read coverage.

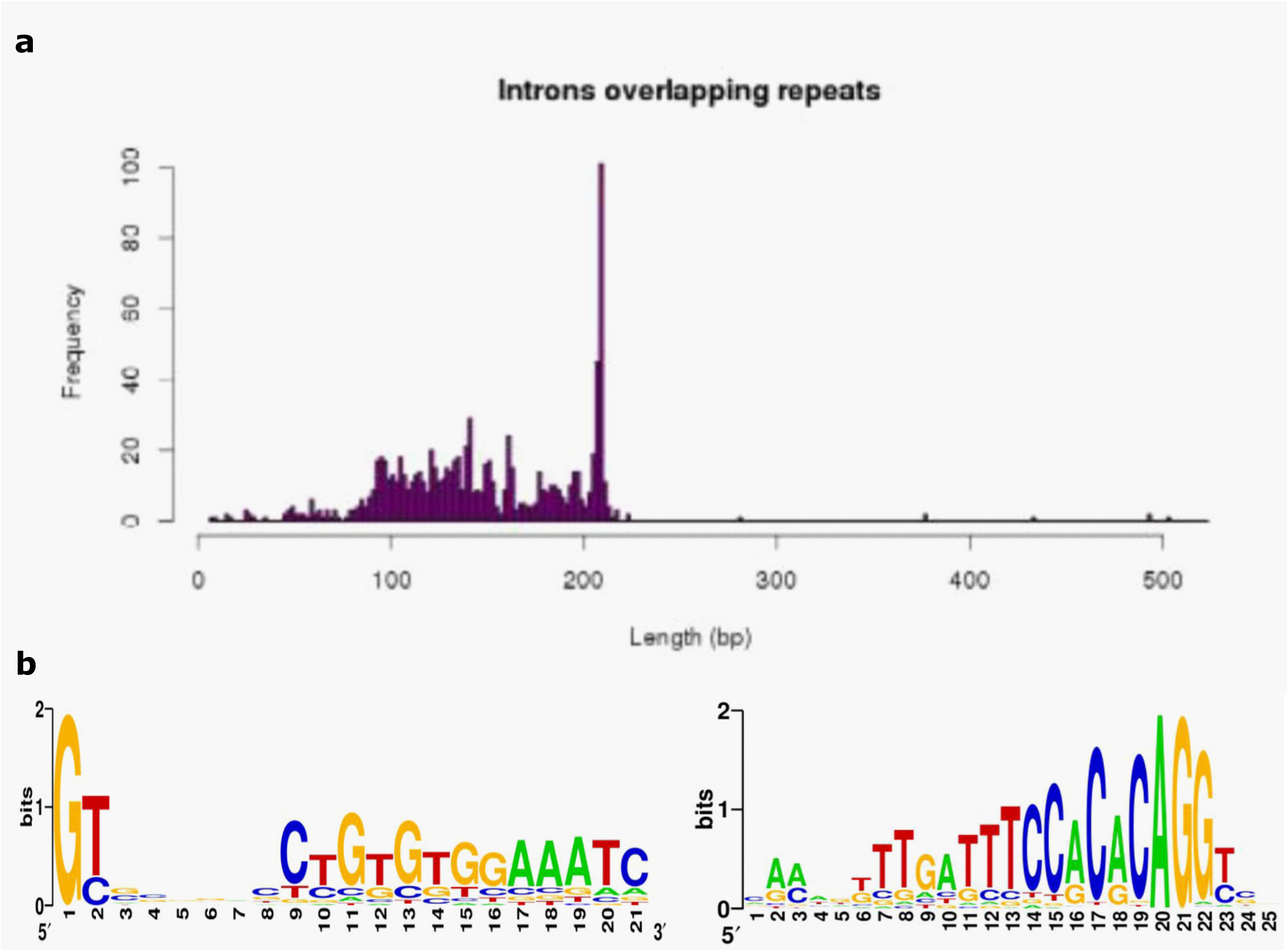

Figure S3: Identification of Introner Elements from repeat families. a) Distribution of putative IE lengths shows a pick around 200 bp. b) Logo representations of the starting and ending sequences of putative IE, revealing the presence of GT or GC donor splicing sites at the 5’ ending, AG acceptor splicing site at the 3’ ending, and conserved Terminal Inverted Repeats at the flanking regions.

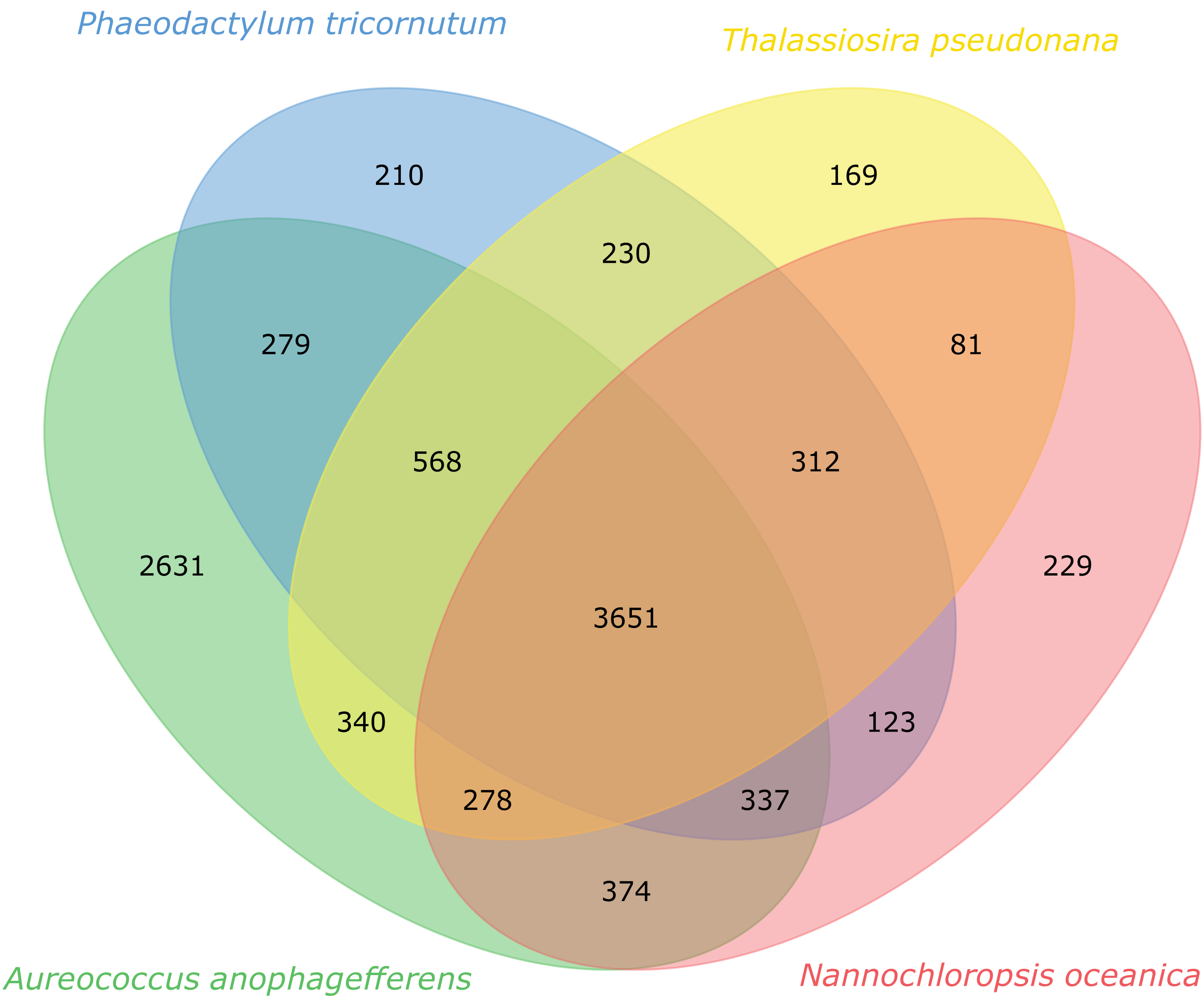

Figure S4: *P. calceolata* proteins shared with other Stramenopiles. The venn diagram displays the number of *P. calceolata* proteins homologous with at least one protein in another Stramenopile genome (alignment length >80% of the shortest protein and evalue $<10^{-5}$).

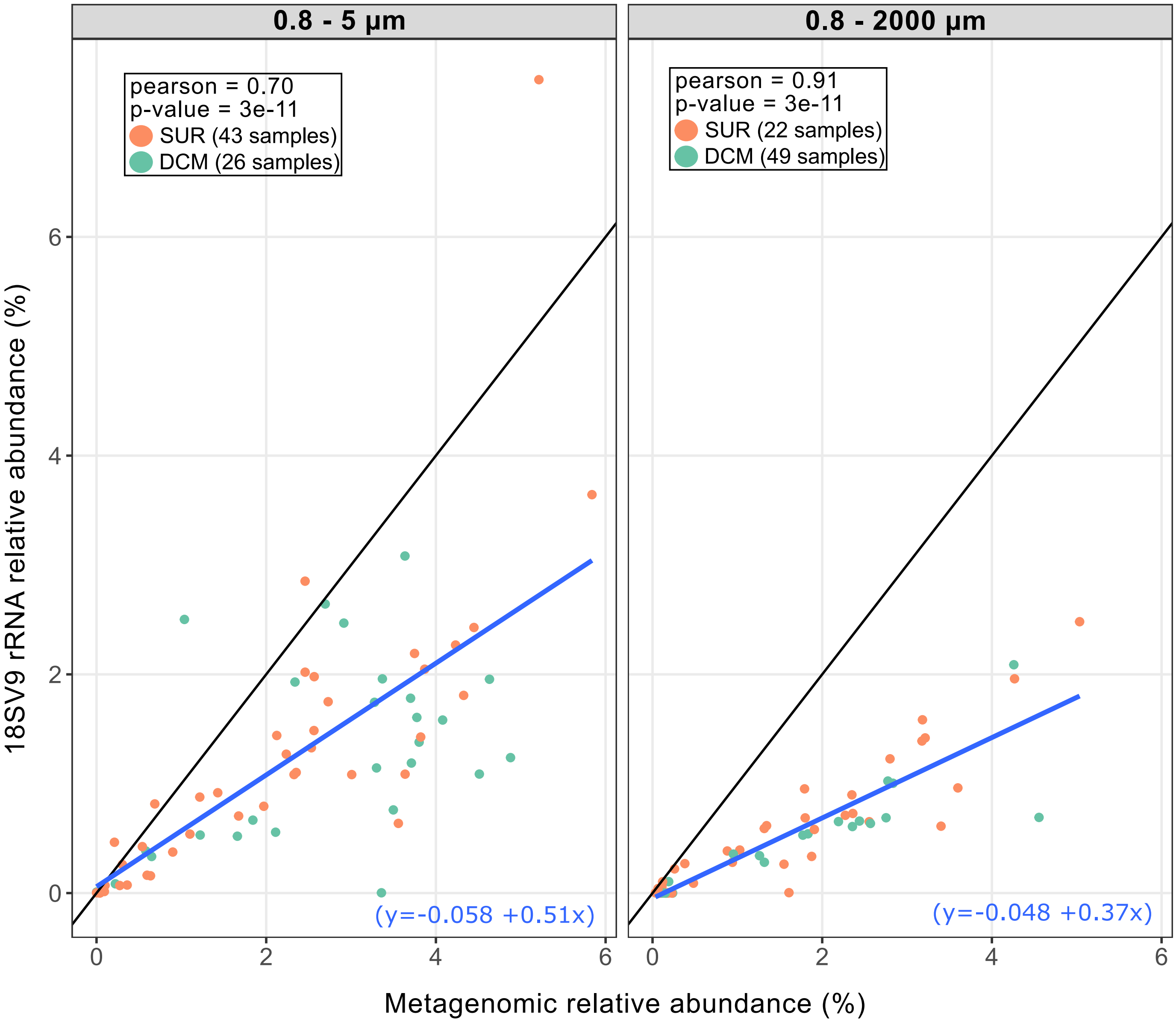

Figure S5: Relative abundance of *P. calceolata* with 2 different methods. Each dot represents a surface (orange) or DCM (green) sample. The relative abundance of *P. calceolata* is calculated from metagenomic reads aligned on the genome (x-axis) and with the amplified 18S rRNA sequence (y-axis) for size-fractions 0.8 - 5 µm (left) and 0.8 - 2000 µm (right). Linear regressions of observed values and the equation are indicated in blue. Black lines are expected trends.

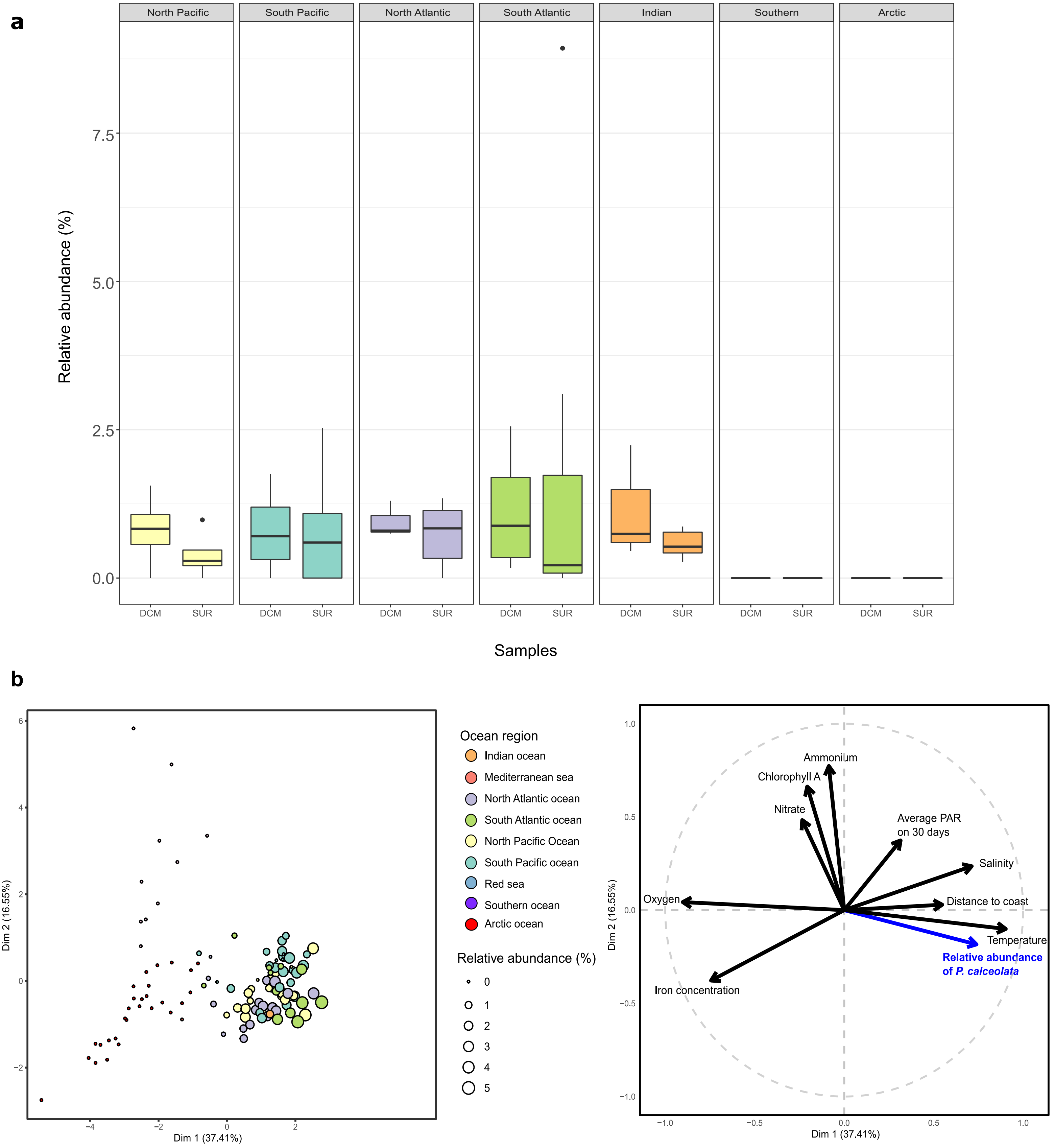

Figure S6: Relative abundance and ecological niche of *P. calceolata* in the 0.8-2000 µm size fraction. a) Boxplot of the relative abundance of *P. calceolata* in each oceanic region in surface and DCM samples in the 0.8 - 2000 µm size-fraction. b) Principal component analysis of the relative abundance of *P. calceolata* and the 9 environmental parameters in the 0.8-2000 µm size fraction. Each dot represents a sample, with a size proportional to the relative abundance of *P. calceolata*. The colours indicate the oceanic basins.

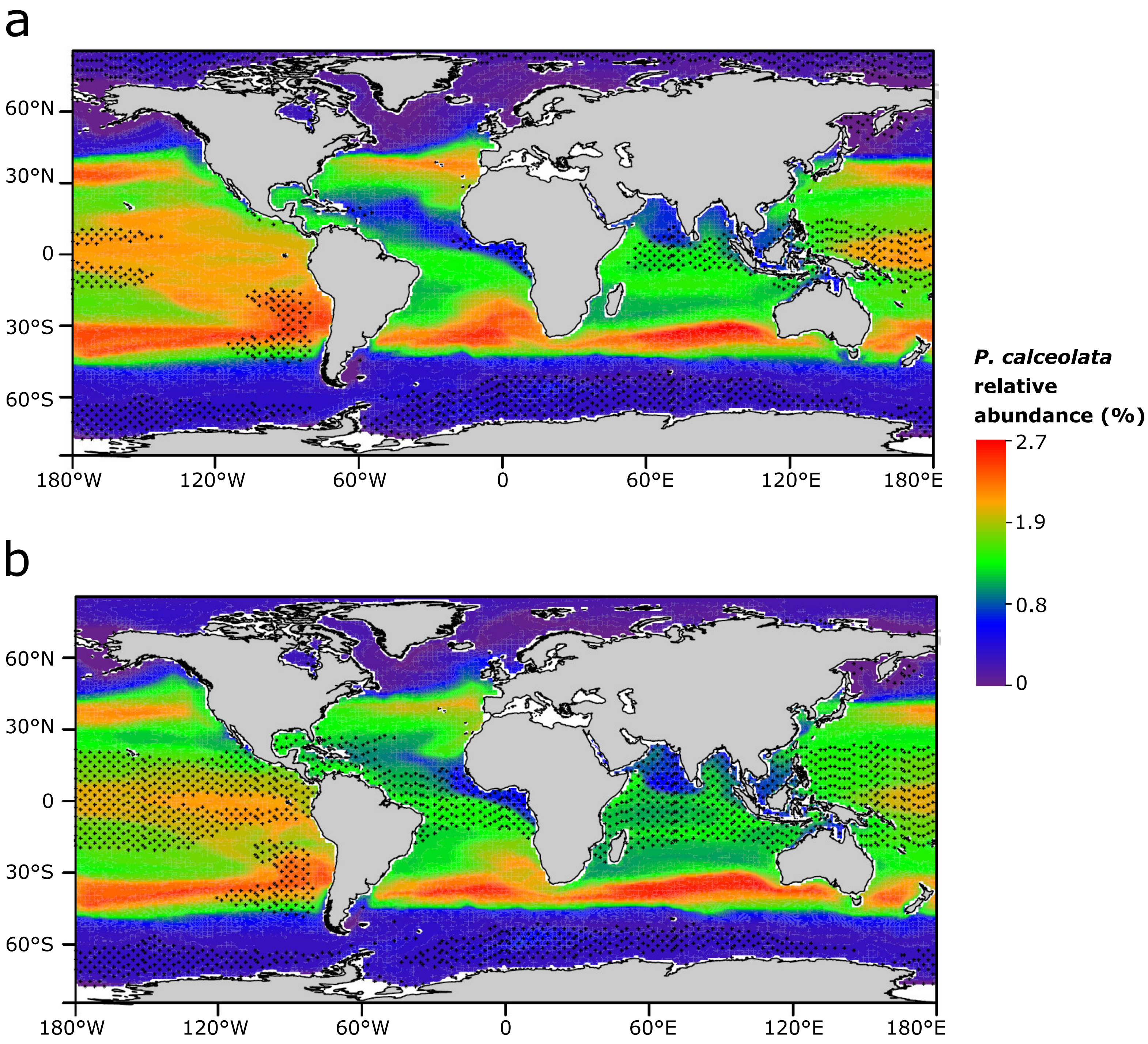

Figure S7: Modelled relative abundance of *P. calceolata* in present day (a) and at the end of the century (b). The relative abundance of *P. calceolata* measured in *Tara* samples (0.8-2000 µm size-fraction) is modeled using the combination of 4 machine learning techniques trained on WOA18 environmental parameters at months, depths and sampling locations of *Tara* Oceans samples (a) or trained on projected climatology in 2099 under the RCP8.5 scenario (b).

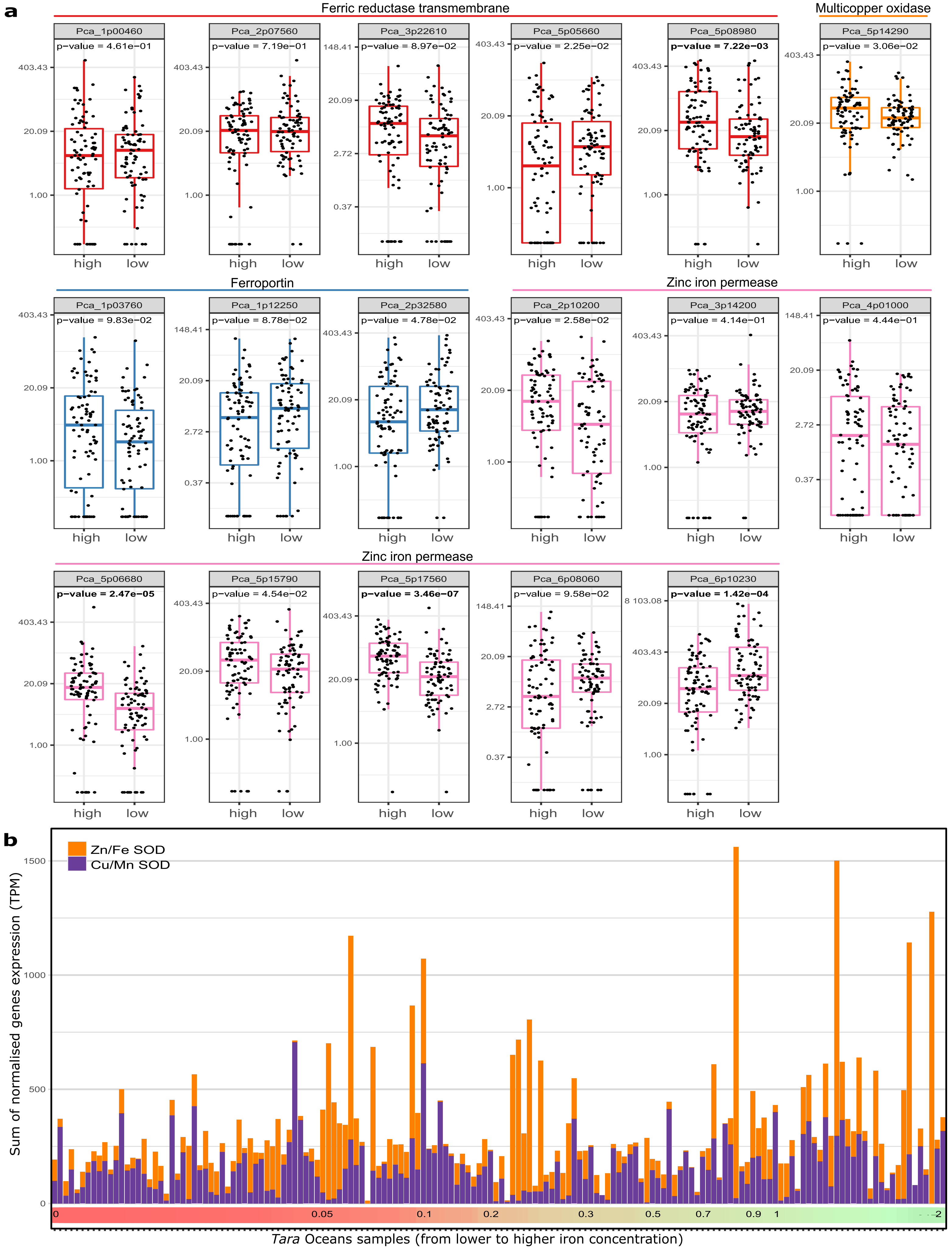

Figure S8: Expression levels of genes involved in iron transport and coding for superoxide dismutases (SOD). a) Relative expression levels (TPM) of genes involved in iron uptake compared between low-iron (<0.2 nM) and high-iron (>0.2 nM) environment. P-values of Wilcoxon statistical tests between low- and high-iron conditions are indicated for each gene. Significant p-values (<0.01) are in bold. b) Relative expression levels (TPM) of genes coding for zinc/iron SOD (orange) and its non-ferrous equivalent copper/manganese SOD (purple) in each *Tara* Oceans sample. Samples are sorted from low-iron (left) to high-iron (right) conditions. Iron concentrations are indicated in nM on the horizontal couloured gradient.

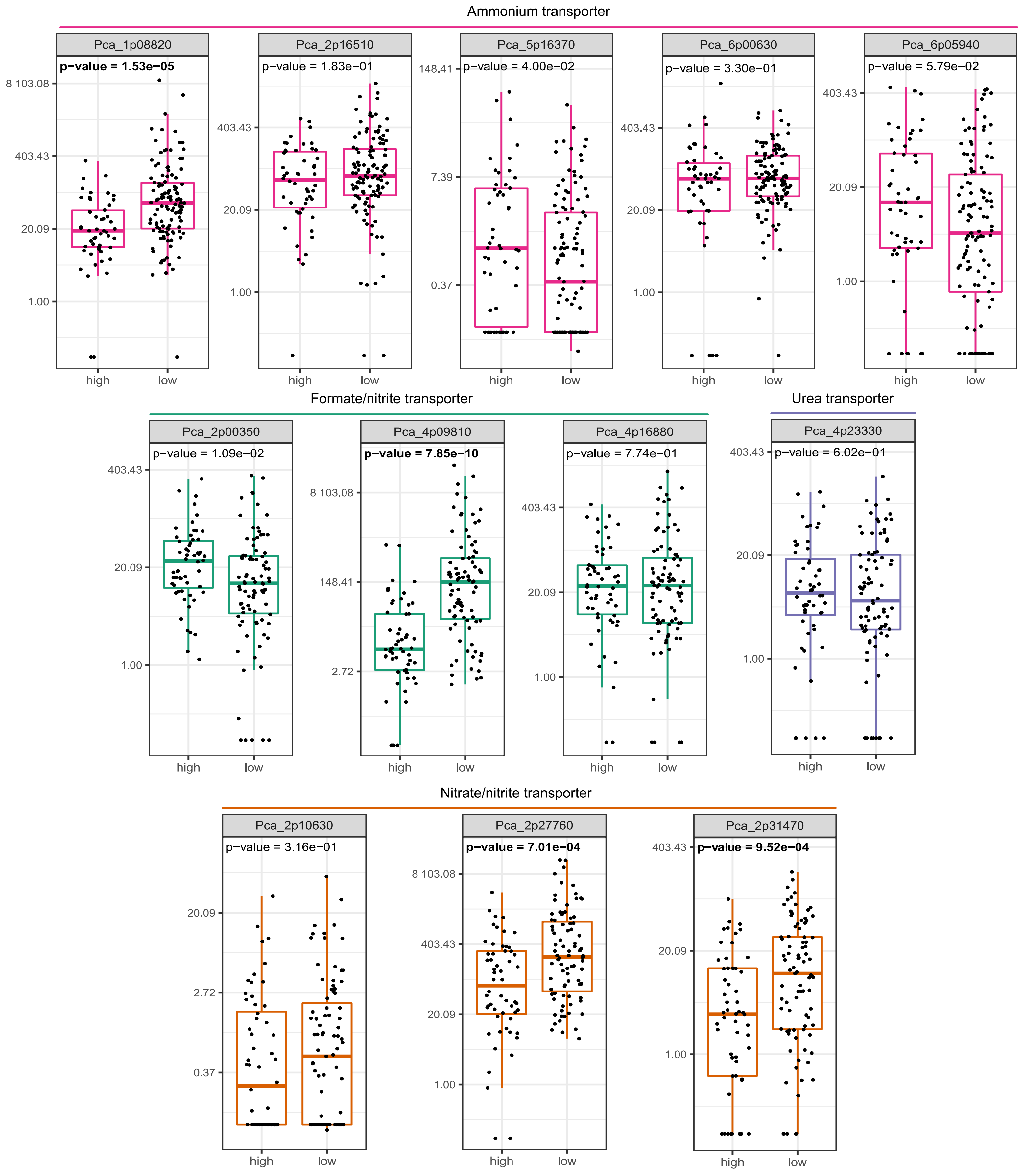

Figure S9: Expression levels of genes involved in nitrogen compounds transport. Relative expression levels (TPM) of genes involved in ammonium uptake compared between low-ammonium (<0.025 µM) and high-ammonium (>0.025 µM) environments, and of genes involved in nitrate, nitrite, formate and urea uptake compared between low-nitrate (<2 µM) and high-nitrate (>2 µM) environments, in the 0.8-5 µm and 0.8-2000 µm size fractions. P-values of Wilcoxon statistical tests between low- and high-ammonuim/nitrate conditions are indicated for each gene. Significant p-values (<0.01) are in bold.

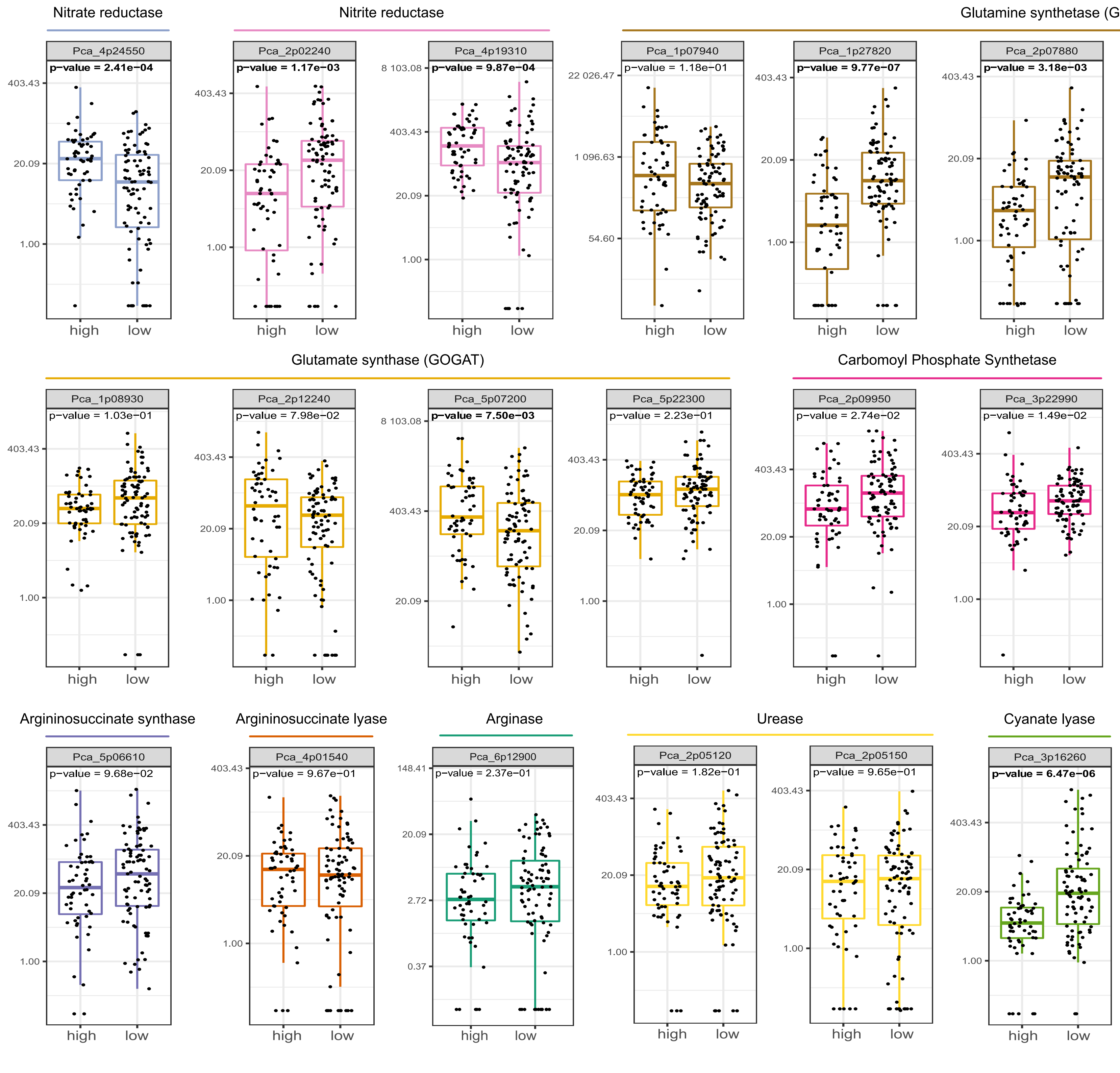

Figure S10: Expression levels of genes involved in nitrogen metabolism. Relative expressions levels (TPM) of genes involved in nitrogen metabolism compared between low-nitrate (<2 μM) and high-nitrate (>2 μM) environments in the 0.8-5 μm and 0.8-2000 μm size fractions. P-values of Wilcoxon statistical tests are indicated for each gene. Significant p-values (<0.01) are in bold.

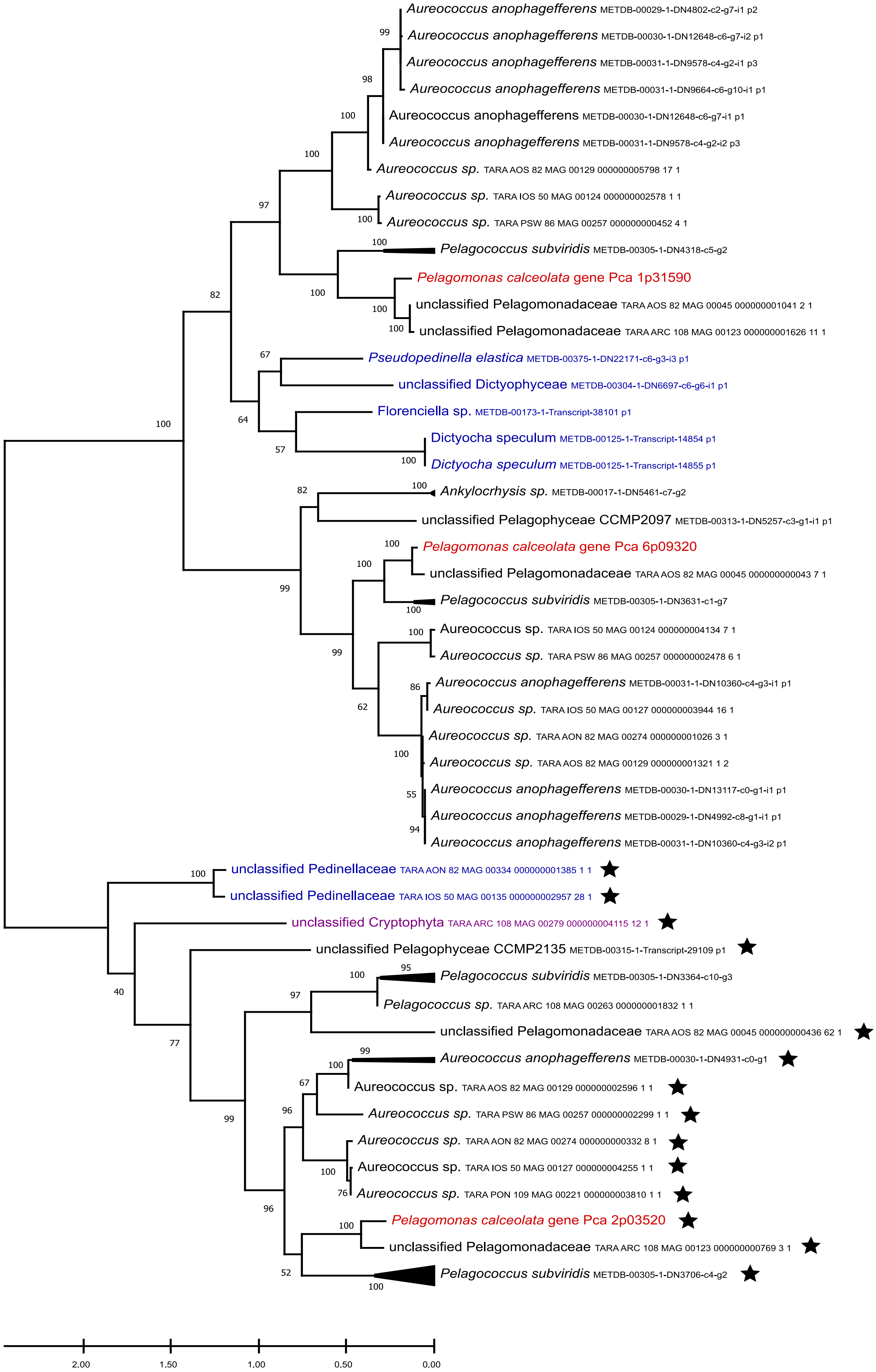

Figure S11: Nitrate and nitrite sensing proteins in *P. calceolata* and other eukaryotes. Maximum likelyhood phylogenetic tree made with JTT (Jones-Taylor-Thornton) model based on multiple alignment of NIT-sensing protein domains homologous to *P. calceolata*. Red: *P. calceolata* sequences. Black: other Pelagophyceae. Blue: Dictyophyceae. Purple: putative Cryptophyceae. The stars indicate NIT-sensing domain surrounded by transmembrane domains.

Supplementary Note 1: Duplicated regions in *P. calceolata* genome

We observed two highly similar duplicated regions (>99% identity) in the *Pelagomonas calceolata* genome. We measured the vertical coverage along the genome to understand if these duplications are real or assembly artefacts. The vertical read coverage of duplicated regions is similar to that of other genomic regions suggesting that they are both present in all cells of this *P. calceolata* culture (Figure 1 and Figure S1d). In addition, 150 Kb at one extremity of contig 4 present a higher vertical coverage suggesting that this region is also duplicated in the *P. calceolata* genome but collapsed in the assembly. Interestingly, duplicate regions at the end of sequences have already been observed in the chlorophyte *Ostreococcus tauri* which has been maintained for several years in culture[1]. These observations suggest that long-time cultures could affect not only the sequence of a given organism's genome but also its structure.

Supplementary Note 2: Description of the Hi-C results

We aligned 75.9% of Hi-C raw reads on the *P. calceolata* genome sequence and nearly 92% of them were considered as Hi-C contacts, showing that the Hi-C preparation was successful. Specific features were observed on the contact map (Figure S2). The red diagonal indicates a large number of contacts within each chromosome arm. In contrast, the number of contacts between arms of the same chromosome is barely above the background. The second striking feature is the unique spot of contacts between all low-GC regions of each chromosome. These contacts confirm the position of the centromeres in each chromosome. Indeed, centromeres co-localised in the nucleus architecture[2] and the Hi-C method is able to reve[2]al this proximity[3,4]. Interestingly, weak contacts were observed between all centromeres and the end of chromosome 3. This is potentially because this region also encompass the repeats detected in the centromeres (Figure 1). In addition, two duplicated regions in the extremities of contigs 1, 3, 5 and 6 present a high number of contact. This is probably an artefact due to the random alignment of these reads in the duplicated regions and do not reflect a specific spatial proximity in the nucleus for these regions. Finally, the beginning of contig 4 present a higher contact background compare to other genomic regions. This is because this region is probably duplicated in the genome but merged in the assembly. Overall, the Hi-C analysis confirms the assembly of *P. calceolata* genome based on ONT long reads.

Supplementary Note 3: Introns and introner elements

The presence of introner elements (IE) in *P. calceolata* was investigated by overlapping the position of the introns in transcriptome alignments with the positions of repeat families detected *ab initio* with RepeatScout[5]. Intron overlapping repeats over more than 90% of their length were identified as

putative IEs. The *P. calceolata* genome contains 956 putative IEs. The logo representation of the putative IE sequences reveals the presence of GT and GC donor sites at the 5' end, AG acceptor site at the 3' end, and conserved TIR in the flanking regions (Figure S3b and Table S3).

Supplementary Note 4: GC content distribution along *P. calceolata* chromosomes

The chromosome level assembly of the *P. calceolata* genome obtained with ONT and Hi-C sequencing revealed unique genomic features that are not usually studied in short-read assembled genomes. The most striking observation is the presence of a unique GC trough (52%) in each putative chromosome contrasting with the high GC content (63%) of other genomic regions of *P. calceolata* suggesting that these low-GC regions encompass centromeres. In eukaryotes, centromeres have a large variety of structures and characteristics. They are composed of many repeated sequences or contain genes, they are determined genetically or epigenetically and their size can vary from 125 bases (in *S. cerevisiae*) to several Mb (in mammals)[6]. Short regional centromeres (1-5 Kb) are generally low-GC compared to the genome and the sequence is unique at each centromere. Among stramenopiles, centromeres were characterized in the diatom *P. tricornatum* where a short low-GC sequence (>500 bp) is enough to define a centromere[7]. Large regional centromeres (>10 Kb) are generally gene-free, contain repeated elements and are not transcribed[6]. *P. calceolata* putative centromeres derive from this general pattern with large low-GC regions containing genes and few repeated elements. Interestingly, the red alga *Cyanidioschyzon* as well as several yeast species seem to have similar centromere structures with large, low-GC centromeres containing genes[8,9].

The gene structure within low-GC regions differs from that of other genomic regions (Figure 1 and Table S5). The slight decrease of gene density observed in Figure 1 is explained by a longer gene size (average of 2.8 Kb compared to 1.7 Kb in other regions). In addition, the 453 genes in low-GC regions contain more introns (2.2 introns per gene compared to 0.4) and the introns are shorter (119 bp compared to 214 bp). We also noticed a higher proportion of bases belonging to intergenic regions, 18% in low-GC regions and 11% in other chromosomic regions (Table S5). Interestingly, three repeats of more than 500 bases are present in several low-GC regions (R_13 in contigs 2, 3 and 6; R_25 in all contigs except contig 1 and R_80 in contigs 1 to 4; see Figure 1). No homology was found between these sequences and known repeat elements. Gene function analysis of low-GC regions reveals an enrichment of genes involved in specific cellular mechanisms (Table S6). Thirteen genes are involved in DNA replication including the Anaphase-promoting complex subunit 4, the sister chromatid cohesion protein Dcc1 and 3 Mini-chromosome maintenance genes. Twenty-five genes are involved in microtubule synthesis and microtubule-binding motor proteins (9 genes carrying dynein domains, 6 genes carrying kinesin motor domains and 4 tubulin genes). These genes indicate that the low-GC

regions contain many genes required for *P. calceolata* cellular division. Finally, 18 genes are involved in transcription including 3 genes encoding RNA Pol II rpb2 subunits and 7 genes encoding transcription factors suggesting an important role of these chromosomic regions for the regulation of gene expression.

Supplementary Note 5: Centromeres, recombination and meiosis in *P. calceolata*

Among 23 gene functions specifically involved in meiosis, 18 homologs are present in the *P. calceolata* genome (Table S7). We did not identified homologs of ZIP, HOP1 and RED1 genes in the *P. calceolata* genome. These genes are known to be involved in homologous pairing of chromatids and construction of the synaptonemal complex in animals, plants and fungi but are not essential to perform meiosis. Indeed these 3 genes are absent in several phyla capable of meiosis like diatoms and ciliates[10,11]. Interestingly, MSH4 and MSH5 genes are absent from the *P. calceolata* genome. These genes necessary to perform the interfering (class I) recombination pathway through Double Holliday Junctions are present in most eukaryotic lineages. The large low-GC regions could be a consequence of the absence of the MSH4/5 genes. Indeed, the main hypothesis to explain these low-GC patterns in centromeres is the importance of GC-biased gene conversions (gBGC) during recombination and the inhibition of this recombination near centromeres[12]. gBGCs increase the GC content of recombining DNA over evolutionary time inducing GC content variations within and between genomes[13]. The kinetochore formation at centromeric regions inhibits recombination and double strand break formation during meiosis resulting in rare gBGC in these regions[14]. Centromeric and peri-centromeric regions may therefore have a lower GC content. In yeasts (*Yarrowia lipolytica*, *Candida lusitaniae*, and *Pichia stipites*), a correlation has been observed between the importance of the GC trough near the centromeres (>10%) and the absence of MSH4/MSH5 genes[9]. It is therefore possible that the absence of this recombination pathway in *P. calceolata* induces more frequently double-strand break repair by synthesis-dependent strand annealing and a more rapid gBGCs across the genome except at the centromeres where double-strand breaks are inhibited. This recombination inhibition may have important consequences on the evolution of the *P. calceolata* genome. Genes within low-GC regions are significantly longer and contain more introns than genes in other genomic regions. Because intron gain and loss are closely related to double-strand break repair and homologous recombination, we suggest that centromere genes retain more introns because double-strand breaks are reduced[15]. Variant analysis in *P. calceolata* populations could be targeted specifically in future studies to infer an estimation of recombination rate and more generally characterize the evolutionary processes controlling these large centromere regions.

In addition, we used the mRNA extracted and sequenced during exponential growth of *P. calceolata* to estimate the relative expression level of meiosis genes. Four out of 18 genes were not detected including the double-strand DNA break initiator SPO11 (Table S7). This result suggests that *P. calceolata* do not perform meiosis in exponential growth.

Supplementary References

1. Blanc-Mathieu, R. *et al.* An improved genome of the model marine alga Ostreococcus tauri unfolds by assessing Illumina de novo assemblies. *BMC Genomics* **15**, 1103 (2014).
2. Mizuguchi, T. *et al.* Cohesin-dependent globules and heterochromatin shape 3D genome architecture in S. pombe. *Nature* **516**, 432–435 (2014).
3. Varoquaux, N. *et al.* Accurate identification of centromere locations in yeast genomes using Hi-C. *Nucleic Acids Res* **43**, 5331–5339 (2015).
4. Muller, H., Gil, J. & Drinnenberg, I. A. The Impact of Centromeres on Spatial Genome Architecture. *Trends in Genetics* **35**, 565–578 (2019).
5. Price, A. L., Jones, N. C. & Pevzner, P. A. De novo identification of repeat families in large genomes. *Bioinformatics* **21 Suppl 1**, i351-358 (2005).
6. Talbert, P. B. & Henikoff, S. What makes a centromere? *Experimental Cell Research* **389**, 111895 (2020).
7. Diner, R. E. *et al.* Diatom centromeres suggest a mechanism for nuclear DNA acquisition. *PNAS* **114**, E6015–E6024 (2017).
8. Kanesaki, Y., Imamura, S., Matsuzaki, M. & Tanaka, K. Identification of centromere regions in chromosomes of a unicellular red alga, Cyanidioschyzon merolae. *FEBS Letters* **589**, 1219–1224 (2015).
9. Lynch, D. B., Logue, M. E., Butler, G. & Wolfe, K. H. Chromosomal G + C content evolution in yeasts: systematic interspecies differences, and GC-poor troughs at centromeres. *Genome Biol Evol* **2**, 572–583 (2010).

10. Chi, J., Mahé, F., Loidl, J., Logsdon, J. & Dunthorn, M. Meiosis gene inventory of four ciliates reveals the prevalence of a synaptonemal complex-independent crossover pathway. *Mol Biol Evol* **31**, 660–672 (2014).

11. Patil, S. *et al.* Identification of the meiotic toolkit in diatoms and exploration of meiosis-specific SPO11 and RAD51 homologs in the sexual species Pseudo-nitzschia multistriata and Seminavis robusta. *BMC Genomics* **16**, 930 (2015).

12. Nambiar, M. & Smith, G. R. Repression of harmful meiotic recombination in centromeric regions. *Semin Cell Dev Biol* **54**, 188–197 (2016).

13. Pessia, E. *et al.* Evidence for Widespread GC-biased Gene Conversion in Eukaryotes. *Genome Biol Evol* **4**, 675–682 (2012).

14. Vincenten, N. *et al.* The kinetochore prevents centromere-proximal crossover recombination during meiosis. *eLife* **4**, e10850 (2015).

15. Farlow, A., Meduri, E. & Schlötterer, C. DNA double-strand break repair and the evolution of intron density. *Trends Genet* **27**, 1–6 (2011).
