# Supplementary material for: Genomic adaptation of the picoeukaryote Pelagomonas calceolata to iron-poor oceans revealed by a chromosome-scale genome sequence
Source: Commun Biol. 2022 Sep 16;5:983. doi: 10.1038/s42003-022-03939-z (PMC9481584; doi:10.1038/s42003-022-03939-z)
Supplement: Supplementary file 3 — Description of Additional Supplementary Files [file 42003_2022_3939_MOESM3_ESM.pdf]

Description of Additional Supplementary Files

File name: Supplementary Data 1
Description: Assembly metrics issued by state-of-the-art tools for de novo genome assembly using long reads. For each assembler, the metrics of 3 separate runs are reported, based on the all sets (All), the longest (Longest) and the best quality (Filtlong) ONT long reads.

File name: Supplementary Data 2
Description: Functional annotation of *P. calceolata* genes

File name: Supplementary Data 3
Description: Introns overlapping repeat. Metrics of introns overlapping the repeats detected ab initio using RepeatScout. For 15% of the introns, the repeat represents more than 90% of the intron length.

File name: Supplementary Data 4
Description: Detailed BUSCO (v 5.0.0) results on *P. calceolata* genome. The lineage dataset used is: stramenopiles_odb10 (Creation date: 2020-08-05, number of species: 27, number of BUSCOs: 100)

File name: Supplementary Data 5
Description: Gene structure in low-GC regions and other genomic regions.

File name: Supplementary Data 6
Description: Pfam domain enrichment in genes present in low-GC regions compared to other chomosomic regions. Pfam domain with adjested p-value < 0.01 are presented.

File name: Supplementary Data 7
Description: Putative meiotic genes identified in *P. calceolata* genome. Kegg Orthology p-values with a star are above the HMM threshold defined by KoFamKoala tool. Their expression levels in exponential growth are indicated in the two last columns of the table.

File name: Supplementary Data 8
Description: 10 most abundant 18SV9 rRNA OTUs in average of all Tara Oceans samples of the 0.8 - 5 µm size-fraction. The average abundance per oceanic layer is provided (SUR=Surface, DCM=Deep-chlorophyll maximum, MES=Mesopelagic).

File name: Supplementary Data 9
Description: Genes involved in nitrogen and iron metabolisms in the *P. calceolata* genome compared to other small photosynthetic eukaryotes.

File name: Supplementary Data 10
Description: *P. calceolata* relative abundance in each sample and associated environmental parameters.
