# Supplementary material for: Genomic adaptation of the picoeukaryote Pelagomonas calceolata to iron-poor oceans revealed by a chromosome-scale genome sequence
Source: Commun Biol. 2022 Sep 16;5:983. doi: 10.1038/s42003-022-03939-z (PMC9481584; doi:10.1038/s42003-022-03939-z)
Supplement: Supplementary file 5 — Reporting Summary [file 42003_2022_3939_MOESM5_ESM.pdf]

nature research

Corresponding author(s): Carradec Quentin

Last updated by author(s): Aug 9, 2022

Reporting Summary

Nature Research wishes to improve the reproducibility of the work that we publish. This form provides structure for consistency and transparency in reporting. For further information on Nature Research policies, see our Editorial Policies and the Editorial Policy Checklist.

Statistics

For all statistical analyses, confirm that the following items are present in the figure legend, table legend, main text, or Methods section.

n/a	Confirmed	
☐	☒	The exact sample size (*n*) for each experimental group/condition, given as a discrete number and unit of measurement
☐	☒	A statement on whether measurements were taken from distinct samples or whether the same sample was measured repeatedly
☐	☒	The statistical test(s) used AND whether they are one- or two-sided<br>*Only common tests should be described solely by name; describe more complex techniques in the Methods section.*
☒	☐	A description of all covariates tested
☐	☒	A description of any assumptions or corrections, such as tests of normality and adjustment for multiple comparisons
☐	☒	A full description of the statistical parameters including central tendency (e.g. means) or other basic estimates (e.g. regression coefficient) AND variation (e.g. standard deviation) or associated estimates of uncertainty (e.g. confidence intervals)
☐	☒	For null hypothesis testing, the test statistic (e.g. *F*, *t*, *r*) with confidence intervals, effect sizes, degrees of freedom and *P* value noted<br>*Give P values as exact values whenever suitable.*
☒	☐	For Bayesian analysis, information on the choice of priors and Markov chain Monte Carlo settings
☒	☐	For hierarchical and complex designs, identification of the appropriate level for tests and full reporting of outcomes
☐	☒	Estimates of effect sizes (e.g. Cohen's *d*, Pearson's *r*), indicating how they were calculated

Our web collection on statistics for biologists contains articles on many of the points above.

Software and code

Policy information about availability of computer code

Data collection	Tara Oceans datasets used in this study are already published and codes used to generate these data are available in the original publication cited in the Mat&Met section.
Data analysis	We haven't developped custom algorithms or software in this study. All softwares used in this study are publicly available, the versions and parameters are detailed in the Mat&Met section. All codes for analysis are available upon a request to the corresponding author.

For manuscripts utilizing custom algorithms or software that are central to the research but not yet described in published literature, software must be made available to editors and reviewers. We strongly encourage code deposition in a community repository (e.g. GitHub). See the Nature Research guidelines for submitting code & software for further information.

Data

Policy information about availability of data

All manuscripts must include a data availability statement. This statement should provide the following information, where applicable:

- Accession codes, unique identifiers, or web links for publicly available datasets
- A list of figures that have associated raw data
- A description of any restrictions on data availability

Pelagomonas calceolata genomic and transcriptomic reads, the genome assembly and gene prediction are available at the ENA (EMBL-EBI) website under the accession number PRJEB47931. P. calceolata transcriptomes are available under the accession number PRJEB34158, runs ERR3497221 and ERR3497222. Tara Oceans and Tara Polar Circle metagenomic sequences are archived at the ENA under the following accession numbers: PRJEB9740, PRJEB9691, PRJEB4352 and PRJEB1787.

Field-specific reporting

Please select the one below that is the best fit for your research. If you are not sure, read the appropriate sections before making your selection.

☐ Life sciences ☐ Behavioural & social sciences ☒ Ecological, evolutionary & environmental sciences

For a reference copy of the document with all sections, see nature.com/documents/nr-reporting-summary-flat.pdf

Ecological, evolutionary & environmental sciences study design

All studies must disclose on these points even when the disclosure is negative.

Study description	In this study, we sequenced the genome of Pelagomonas calceolata and used it as a reference to estimate P. calceolata abundance in the oceans and analysed its adaptation capacities. Environmental samples were collected between 2009 and 2013 in the framework of Tara Oceans expedition.
Research sample	Pelagomonas RCC100 strain used to sequenced the genome is maintained in the Roscoff Culture Collection in France (https://roscoff-culture-collection.org/). Tara Oceans samples used in this stuydy as well as environmental parameters measured during the expedition are described in Pesant et al 2015 doi:10.1038/sdata.2015.23). Metagenomic, metatranscriptomic and metabarcoding datasets are described in Alberti et al 2017 (doi:10.1038/sdata.2017.93).
Sampling strategy	Data used in this study are already available so the sample size is fixed. For all statistical analysis the sample size is indicated and we performed comparisons only when the number of samples was adequate.
Data collection	Data collection procedure is detailed in Pesant et al 2015 (doi:10.1038/sdata.2015.23)
Timing and spatial scale	The Tara Oceans expedition (2009–2013) sampled contrasting ecosystems of the world oceans, collecting environmental data and plankton. It surveyed 210 ecosystems in 20 biogeographic provinces, collecting over 35,000 samples of seawater and plankton (Pesant et al 2015).
Data exclusions	Samples containing not enough sequenced reads for P. calceolata were excluded from the study. Criteria of exclusion are indicated in the Mat&Met and are constant along the study.
Reproducibility	The environmental sampling Tara Oceans don't contain replicates. We used the large number of samples collected to perform our statistical tests.
Randomization	Not relevant for this environemental study.
Blinding	Not relevant for this environemental study.

Did the study involve field work? ☒ Yes ☐ No

Field work, collection and transport

Field conditions	Data already published and described in Pesant et al 2015
Location	Data already published and described in Pesant et al 2015
Access & import/export	Data already published and described in Pesant et al 2015
Disturbance	Data already published and described in Pesant et al 2015

Reporting for specific materials, systems and methods

We require information from authors about some types of materials, experimental systems and methods used in many studies. Here, indicate whether each material, system or method listed is relevant to your study. If you are not sure if a list item applies to your research, read the appropriate section before selecting a response.

Materials & experimental systems

n/a	Involved in the study
☒	☐ Antibodies
☐	☒ Eukaryotic cell lines
☒	☐ Palaeontology and archaeology
☒	☐ Animals and other organisms
☒	☐ Human research participants
☒	☐ Clinical data
☒	☐ Dual use research of concern

Methods

n/a	Involved in the study
☒	☐ ChIP-seq
☒	☐ Flow cytometry
☒	☐ MRI-based neuroimaging

Eukaryotic cell lines

Policy information about cell lines

Cell line source(s)	P. calceolata RCC100 is maintained at the Roscoff Culture Collection : https://roscoff-culture-collection.org/
Authentication	The 18S rRNA sequence was sequenced to confirm the identification of the cell culture.
Mycoplasma contamination	The cell line was not tested for mycoplasma contamination.
Commonly misidentified lines (See ICLAC register)	None
